# Iron Oxide Powder as Responsible for the Generation of Industrial Polypropylene Waste and as a Co-Catalyst for the Pyrolysis of Non-Additive Resins

**DOI:** 10.3390/ijms231911708

**Published:** 2022-10-03

**Authors:** Joaquín Hernández-Fernández, John R. Castro-Suarez, Carlos A. T. Toloza

**Affiliations:** 1 Chemistry Program, Department of Natural and Exact Sciences, San Pablo Campus, University of Cartagena, Cartagena 130015, Colombia; 2 Chemical Engineering Program, School of Engineering, Universidad Tecnológica de Bolivar, Parque Industrial y Tecnológico Carlos Vélez Pombo Km 1 Vía Turbaco, Cartagena 130001, Colombia; 3 Área Básicas Exactas, Universidad del Sinú, Seccional Cartagena, Cartagena 130015, Colombia; 4 Department of Natural and Exact Science, Universidad de la Costa, Barranquilla 080002, Colombia

**Keywords:** iron oxide, pyrolysis, industrial waste, co-catalysis, polypropylene

## Abstract

For the synthesis of polymeric resins, it is of great importance to review the raw materials and the equipment to be used to avoid the presence of compounds that may affect the effectiveness of the polymerization and the characteristics of the plastic to be obtained. Iron oxide is a compound that can be present in reactors after maintenance due to the techniques used and the cleaning of this equipment, and it can affect the characteristics of the resins, reducing their quality. In this study, the presence of FeO in different concentrations was evaluated to determine its effects on the properties and pyrolysis of polypropylene resins by using X-ray refraction to determine the elements of the samples, evaluating thermal degradation by TGA, the variation in molecular weight by measuring the MFI, and the compounds obtained from pyrolysis by chromatography. The results showed that the thermal degradation decreased as the FeO concentration increased, while for the MFI, the relationship was directly proportional. The evaluation of the compounds obtained from pyrolysis showed an increase in the production of alcohols, alkynes, ketones, and acids, and a decrease in alkanes and alkenes, showing that FeO affects the properties of polypropylene and the compounds that are produced during pyrolysis.

## 1. Introduction

To meet the high demand for plastic materials such as polypropylene, companies have commented on their production volume, generating large amounts of materials that will probably end up as solid waste [1,2]; However, during the production of the polymers, failures or errors occur that can produce materials that do not meet the required characteristics or standards, but even so, they can be sold as materials by specifications [3]. If the material cannot be sold or has characteristics that make it difficult to implement in other processes, it is incinerated, a process that can generate contaminants from the thermal degradation of the material.

Due to the fact that incineration is a common practice to treat resins that are not commercialized or that are residues, the effects of the incineration atmosphere (oxidative or inert) and the presence of compounds in the catalysts (aluminas, zeolites, silica), which may affect the kinetics of the thermal degradation of polymeric materials, the way in which catalysts can be obtained that allow for degradation to be accelerated, and improvements in the performance of the pyrolysis used for the revaluation of polymeric material waste [4,5,6,7,8,9]. The understanding of the degradation kinetics of polymers allows one to obtain parameters that will be used to establish operating conditions, resin treatments, and waste recovery processes. This is established by studying the synthesis processes of polymeric materials such as polypropylene, which for its synthesis uses Ziegler Natta-type catalysts based on titanium tetrachloride with magnesium chloride support and uses organometallic co-catalyst systems such as triethylaluminum (TEAL) [4,10,11,12,13]. This amount of catalysts and catalysts can generate traces of the compounds that compose it in the generated polymeric matrix, and, in turn, these can generate oxidative effects when combined with some residues such as iron oxide (FeO), generating volatile compounds [14,15,16]. However, the recording kinetics of this interaction has been studied very little, focusing a little more on the prediction of the compounds that can be formed during the pyrolysis of the resin [17,18,19].

With the aim of implementing techniques that allow industrially generated waste to be revalued, thermal and catalytic recording have been studied for the generation of excellent quality products that can be marketed [20]. However, it is necessary to understand how compounds such as iron oxide can affect the production of polypropylene, generating waste as well as how the autocatalytic thermal degradation of these wastes would affect pyrolysis [3,21,22,23,24,25]. It is important to evaluate the presence of iron oxide in the synthesis and autocatalysis of polypropylene, since during the preventive and corrective maintenance of petrochemical plants that are dedicated to the synthesis, production, and commercialization of polypropylene, traces of FeO can be released by cleaning the internal walls of the reactors with high-pressure sand and washing with process water that favors the detachment of trace metals from carbon steel. Due to the fact that the subsequent washes are not 100% efficient, some traces of iron remain inside the reactor. In this research, different levels of the concentration of FeO spheres in polypropylene were evaluated to study the effects on the final properties of the fluidity of PP, its thermal degradation, and its incidence in the autocatalytic pyrolysis of polypropylene, applying gas chromatography to determine and quickly and simultaneously quantify the oxygenated compounds, hydrocarbons, and permanent gases formed by the effects of iron oxide beads.

## 2. Results and Discussion

### 2.1. Elemental Analysis of PP Samples

Each of the samples obtained was evaluated to determine the characteristic compounds of each through an analysis where it was determined that the samples contained humidity between 0.21 and 0.42%. The presence of elements such as carbon (>83%) and hydrogen (between 14.05 and 14.82%) in greater proportion due to the polymeric chain of PP, in addition to compounds such as nitrogen, sulfur, aluminum, chlorine, and titanium from the catalysts and catalysts used during the synthesis of the resin, and the presence of added iron oxide was confirmed in the amounts of 4.13, 15, 121, 227, and 351 mg kg^−1^, as shown in Table 1.

Through this analysis, it was also possible to show that the iron content did not significantly affect the concentrations of each of the characteristic elements of polypropylene. However, its presence may indicate adverse effects on some properties of the material and that, under different concentrations and conditions of work, can react with some elements present such as the residual metals of the catalysts and catalysts used during pyrolysis, affecting its performance.

### 2.2. MFI

**Figure 1:**
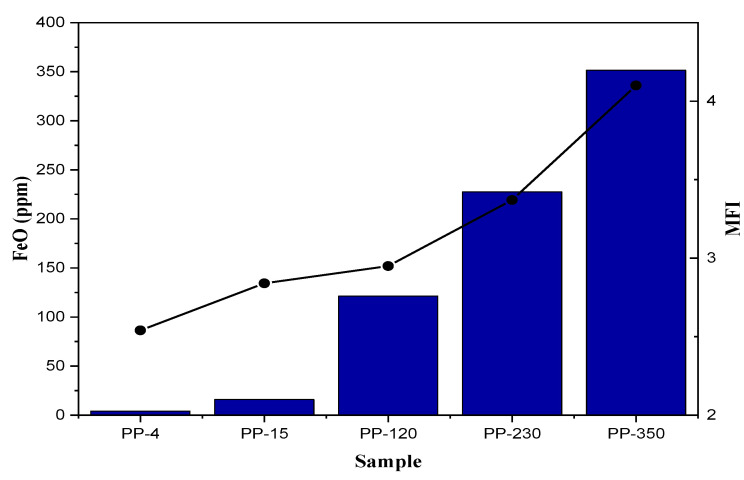
The relationship between the concentration of iron oxide and the MFI of PP.

Because the MFI is related to the average molecular weight, the fluidity index can tell whether the studied material’s molecular properties have changed. In this study, it was found that as the concentration of iron oxide present in the resin samples increased, the MFI also increased, indicating a relationship between them. The significant increase in the MFI indicates the future effects on some properties of the material that may affect the effectiveness of pyrolysis. Figure 1 shows the relationship between the MFI and the content of iron oxide in the PP, showing an increase of more than 60% in the highest concentration, and for the lowest concentration (4.15 ppm), there was no increase in the MFI, indicating that as long as the concentration of iron oxide does not exceed 4 ppm, it will not affect the molecular weight of the material obtained. The increase in the MFI when evaluated with the elemental analysis of the samples shows that this increase is essentially due to the cleavage of the chain and not to oxidation, as has been evidenced in other studies [26,27,28].

The increase in MFI in the samples of polymers synthesized in the presence of external compounds has been evaluated by various authors, where it has been determined that this phenomenon occurs due to the interaction between the ZN catalyst and the different compounds that may be present during polymerization (poisons or external donors), causing a decrease in the molecular weight of the resin obtained [25,29,30,31], which translates into an increase in the MFI according to the relationship between the average molecular weight and the MFI proposed by Bremner [32,33].

### 2.3. TGA

**Figure 2:**
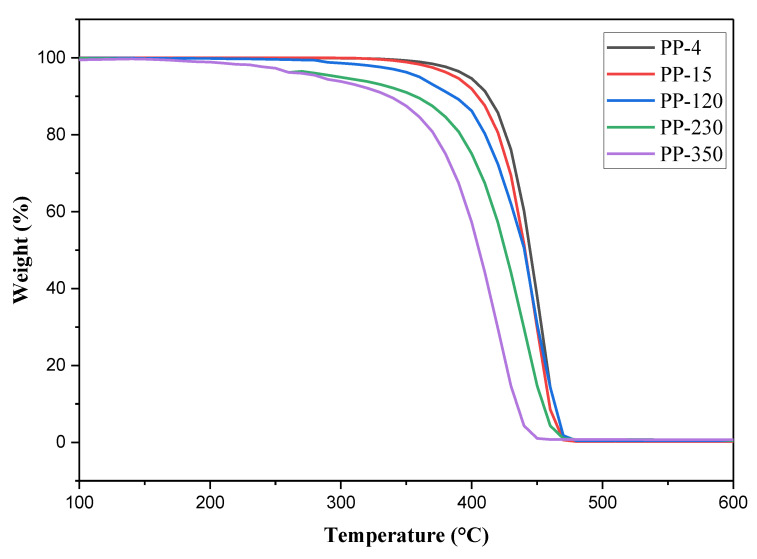
The thermogravimetric analysis of the PP samples.

The thermal degradation of the resins obtained was evaluated by thermogravimetric analysis, as shown in Figure 2, where the incidence of iron oxide on material degradation is evident. This analysis also demonstrates how the temperature range at which the resin’s mass decreases shift, with the lowest concentration of iron oxide having a range of mass loss varying between 270 and 540 °C, while the resin with the highest iron oxide content had a degradation that began at 100 °C and stopped losing mass at 580 °C. At the temperature where they lost 50% of the mass, it was determined that it decreased as the concentration of iron oxide increased, obtaining the highest concentration of FeO when the mass was reduced to 50% when it reached a temperature of around 414 °C, while the same percentage of weight loss was reached at around 450 °C for the lowest concentration.

The effects of iron on the synthesis of resins have been studied very little and it has been focused mainly on the synthesis of polymeric nanocomposites of high-density polyethylene, where the thermal degradation of the material presents positive changes but not very marked [34,35,36]. This is mainly due because other support compounds such as starch are used to guarantee a better distribution of iron on the polymeric matrix and in low concentrations [35]. Unlike these studies, the iron present in the samples presented came from process residues that are not intentional, nor do they present a support that allows for good interaction with the catalysts during the synthesis of polypropylene, which favors a correct distribution of the molecules of the iron oxide so that the molecules are not affected. Therefore, it is necessary to carry out in-depth studies on the polymerization reactions that occur during the synthesis of polypropylene to understand how they affect the resin at a structural level, which causes the change in its properties.

### 2.4. Characterization of Pyrolysis Gaseous and Liquid Products

Through chromatography and mass spectrometry, the different compounds can be determined that can be grouped into six main functional groups to evaluate which functional group was produced in greater quantity and which less. The compounds obtained were classified into alkanes, alkenes, alkynes, ketones, alcohols, and acids. The compounds belonging to each group were obtained by mass spectrometry. Ethyne (C_2_H_2_), ethylene (C_2_H_4_), and propane (C_3_H_8_) could be determined by fragments *m*/*z* 26, 28, and 44, and fragments *m*/*z* 50, 52, 54, and 56 belong to C4 compounds such as 1,3-butadiino (C_4_H_2_), vinyl acetylene (C_4_H_4_), 1,3-butadiene [37], and 1-butene or 2-methylpropene. With evidence of diagnostic elements of *m*/*z* 62, 63, 67, 68, 69, and 71, the *m*/*z* ratios of 65, 70, and 72 were attributed to cyclopentane or methyl butene and pentane. Meanwhile, during the thermal decomposition of other plastic waste, oxygenated chemicals such as acetic acid (*m*/*z* 59, 60) and propionic acid (*m*/*z* 59, 60, 73, 74) were discovered [38].

Table 2 shows the concentrations of the compounds identified in each of the PP resins. Among the alkanes were the following compounds: methane, ethane, propane, cyclopropane, isobutane, N-butane, and isopentane. A higher proportion of ethane with concentrations between 12.4 and 1.4% mol and isopentane with concentrations between 12.1 and 5.4% mol were obtained. In the case of the alkane group, it was shown that the total content in molar percentage decreased as the iron oxide content increased, going from 35.35 to 10.77. In the case of alkenes, a similar behavior was shown, where the total content of compounds in this group decreased from 62.6 to 34.55, being the compound with the highest proportion of propylene, which was expected to be due to the nature of the material, obtaining concentrations from 57.1 to 25.75 mol%. Unlike the previous two functional groups, the alkynes increased as the content of iron oxide in the sample increased. In this group, only two acetylene and one methyl acetylene compounds were evident, where the latter was found in a greater proportion.

As for the remaining identified groups (alcohols, ketones, and acids), the same behavior as for the alkynes was observed whereas the concentration of iron oxide increased, the content of these compounds also increased. In the case of alcohols, not only did the total content of these compounds increase, but it also appeared that for concentrations greater than 4 ppm of FeO, N-butyl alcohol, 1.2.-isobutanediol, and 3-methyl-2 begin to be produced and pentanol, with the latter being when the concentration is greater than 15 ppm of FeO. The total concentrations of the functional groups did not allow us to find which ones were found in greater proportion and what trend followed. Organizing the values obtained, we found that they followed the following pattern for PP-4: alkenes > alkanes > alkynes > acids > ketones > alcohols. However, this sequence varied with the FeO concentration due to the fact that the increase in this compound in PP favors the production of alcohols.

The effects of various metal oxides on the pyrolysis of polymeric residues have been widely studied, generally focusing on zinc, titanium, magnesium, and silicon oxides [39,40]. In the case of iron oxides, Fe_2_O_3_, supported with other compounds, is used as a pyrolysis catalyst to obtain aromatic hydrocarbons, concluding that iron influences pyrolysis due to Fe dispersion, surface area, and moderate total acidity [41,42,43]. However, the effects of metal oxides present in the polymeric matrix during pyrolysis have not been reported previously, which indicates the variation in the formation of gaseous and liquid compounds produced by pyrolysis.

## 3. Materials and Methods

### 3.1. Standards

The standards of hydrocarbon oxygenated compounds and permanent gases certified by Airgas USA LLC were used, to which the chromatographic profile and the concentration of the compounds in the balance with helium and propylene were evaluated.

### 3.2. Preparation of FeO and Ziegler Natta Catalyst Mixtures

The impregnation method was used to prepare the Ziegler Natta catalyst mixture with the FeO concentrations of interest. Specifically, 4, 15, 120, 230, and 350 mg of FeO were dissolved in 1000 g of a commercial mix of the Ziegler Natta catalyst and stirred with a magnetic stirrer. The process was carried out in an extraction cabinet for 1 h.

### 3.3. Site of Sampling and Collection of Samples

The manufacturing process mainly involves four stages: (1) Reception, purification, and storage of raw materials; (2) the polymerization procedure; (3) additivation and pelletizing; and (4) odor elimination. The samples of interest in this research were taken at stage 2, just after the polymerization and before the extrusion and additive stages of the PP resins, where five types of polypropylene waste with different concentrations of iron oxide were generated, which were stored sent at high pressure, previously cleaned with high purity. The samples were taken for a period of 6 months at the same point for subsequent size reduction until a 1 mm particle was obtained.

### 3.4. XRF

Metal residues generated during catalysis were analyzed by X-ray fluorescence using a Malvern Panalytical Axios FAST elemental analyzer and Zetium polymer editing elemental analyzer.

### 3.5. MFI

A Tinius Olsen MP1200 Plastometer was used to measure the melt flow index (MFI). The temperature inside the plastometer barrel was 230 °C, and a 2.16 kg piston was used to displace the melt.

### 3.6. TGA

For the thermogravimetric analysis, 10 mg of the samples were placed in crucibles and pyrolyzed with a nitrogen flow, maintaining a constant temperature increase of 20° per minute from 25 °C to 600 °C, obtaining a thermal record for each of the samples.

### 3.7. Pyrolysis

The pyrolysis of PPs (around 20 g per run) was carried out in a quartz reactor installed in a horizontal tube furnace. Before pyrolysis, the residues were analyzed to determine their Fe concentration, humidity, and thermal stability. The pyrolysis was carried out first in a nitrogen atmosphere. A nitrogen flow of 100 mL min^−1^ was continuously maintained to guarantee the inertia of the environment during the tests, which made its way through the reactor. The temperature used for pyrolysis was 600 °C at constant heating rates (10 °C min^−1^). After the completion of pyrolysis, the solid carbonaceous residue was removed from inside the quartz reactor for further examination.

### 3.8. GC-MS/PDHID/FIDA Analysis of Pyrolysis Gases and Condensates

A 7890B gas chromatograph with front and rear split/splitless injector ports was used for characterization and has three detectors and eight columns. The total time of the race was 37.14 min. The flow rate of the helium carrier gas was set at 2.8 mL/min. The vs. are used to carry gas to the columns and, ultimately, to the detectors of interest. To reduce the possibility of occluding the capillary restrictor or damaging the liquid sample rotor, all liquid samples must be filtered. A Nupro “FW” series 2–7 micron in-line filter or equivalent is required.

An Agilent InertPlus 5977 MS quadrupole equipped with an ExtrEITM 350 electronic impact ionization (EI) source was used to identify and quantify oxygen and sulfur compounds. The quadrupole temperature was 150 degrees Celsius, and the transfer line temperature was 280 degrees Celsius.

The solvent delay was 0.0 min, and the fixed energy of the electrons was 70.0 ev. A 170 mm × 0.11 mm fused silica restrictor (Agilent G3185-60362) was installed on the spectrometer to achieve a fixed flow rate of 1.4 mL min^−1^ He. The FID detector (250 °C, 30 mL min^−1^ H_2_, 400 mL min^−1^ air) was used to examine the hydrocarbon family, while the PDHID detector (220 °C) was used to study the permanent gases [44].

## 4. Conclusions

In this study, the effects of iron on the properties of polypropylene such as melt flow rate and thermal degradability of polypropylene resins with different concentrations of this compound are determined. Due to the processes that are carried out to prevent equipment failure, iron oxide may be present during the synthesis of polypropylene resins obtained after the maintenance of the reactors. The presence of this compound not only affects the characteristics of the material, generating resins by specifications that in many cases would go through pyrolysis processes to take great advantage of them. However, the presence of iron oxide encourages the production of alcohols and decreases production. Compounds such as alkenes and alkanes can be very useful for reevaluating these compounds. The results obtained indicate that the iron oxide interacts with the ZN catalyst and enters the polymeric matrix, changing the composition of the resins and affecting the compounds produced by pyrolysis. The foregoing makes it necessary to study in depth the effects that the poisons present during the polymerization have on the materials produced and the revaluation processes.

## Figures and Tables

**Table 1:** Elemental analysis of the PP samples with different concentrations of iron oxide.

Feed	PP-4	PP-15	PP-120	PP-230	PP-350
**Proximate analysis (as received)**					
Moisture, wt.%	0.21	0.37	0.41	0.34	0.42
Volatile matter, wt.%	99.58	99.43	99.42	99.39	99.45
Fixed carbon, wt.%	0.11	0.13	0.13	0.18	0.09
Ash, wt.%)	0.05	0.06	0.04	0.09	0.04
**Ultimate analysis (as received, wt.%)**					
C, wt.%	83.79	83.79	84.15	84.37	83.84
H, wt.%	14.75	14.82	14.18	14.05	14.58
N, wt.%	0.07	0.05	0.09	0.07	0.05
S, wt.%	0.32	0.39	0.41	0.47	0.41
O, wt.%	1.06	0.95	1.18	1.04	1.11
Ti, mg Kg^−1^	0.87	0.87	0.87	0.87	0.87
Al, mg Kg^−1^	9.16	10.11	8.53	9.27	8.45
Cl, mg Kg^−1^	13.43	13.82	13.91	14.17	13.95
FeO, mg Kg^−1^	4.13	15	121	227	351

**Table 2:** The concentrations of the identified pyrolysis compounds.

Compounds	SAMPLES				
	PP-4	PP-15	PP-120	PP-230	PP-350
**ALKANS**					
Methane, % mol	5.84 ± 0.0529	4.13 ± 0.1155	4.57 ± 0.0577	3.38 ± 0.0764	1.37 ± 0.0577
Ethane, % mol	12.33 ± 0.1155	11.80 ± 0.1000	9.38 ± 0.0764	4.67 ± 0.0577	1.44 ± 0.0693
Propane, % mol	3.10 ± 0.10	3.38 ± 0.0681	4.27 ± 0.1155	2.85 ± 0.050	1.17 ± 0.1155
Cyclopropane, % mol	0.05 ± 0.0058	0.57 ± 0.0577	0.24 ± 0.0058	0.21 ± 0.0173	0.07 ± 0.0058
Isobutane, % mol	0.48 ± 0.0764	0.70 ± 0.0058	0.22 ± 0.0289	0.32 ± 0.0289	0.12 ± 0.0289
N-Butane, % mol	1.40 ± 0.10	1.00 ± 0.1732	1.40 ± 0.0	2.18 ± 0.1041	1.33 ± 0.0577
Isopentane, % mol	12.03 ± 0.0577	8.79 ± 0.0503	6.52 ± 0.0289	11.81 ± 0.0173	5.37 ± 0.0577
***Total Amount***	***35.24 ± 0.2873***	***30.37 ± 0.1102***	***26.59 ± 0.0551***	***25.42 ± 0.1825***	***10.86 ± 0.1137***
**ALKENES**					
Ethylene, % mol	1.71 ± 0.0231	0.89 ± 0.0173	1.47 ± 0.1155	1.50 ± 0.0058	1.47 ± 0.1155
Propylene, % mol	57.15 ± 0.05	54.10 ± 0.10	49.60 ± 0.1732	37.81 ± 0.0173	25.76 ± 0.0231
Propadyene, % mol	0.82 ± 0.0153	0.11 ± 0.0115	0.41 ± 0.0115	0.71 ± 0.0115	1.13 ± 0.1155
Trans-2-Butene, % mol	0.21 ± 0.0173	0.22 ± 0.0289	0.40 ± 0.0	0.51 ± 0.0173	1.07 ± 0.0577
1-Butene, % mol	0.91 ± 0.0173	0.23 ± 0.0306	0.61 ± 0.0231	0.71 ± 0.0115	2.09 ± 0.0808
Cis-2-Butene, % mol	0.31 ± 0.0173	0.44 ± 0.0551	0.71 ± 0.0115	0.30 ± 0.0058	0.82 ± 0.0289
1,3-Butadiene, % mol	0.70 ± 0.0058	9.12 ± 0.1258	5.38 ± 0.0289	0.10 ± 0.0012	1.32 ± 0.0346
1-Pentene, % mol	0.91 ± 0.0173	0.81 ± 0.0115	0.49 ± 0.0115	1.80 ± 0.0058	0.90 ± 0.0058
***Total Amount***	***62.64 ± 0.0635***	***65.85 ± 0.1848***	***59.00 ± 0.1674***	***43.41 ± 0.0115***	***34.58 ± 0.0520***
**ALKYNES**					
Acetylene, % mol	0.21 ± 0.0115	0.23 ± 0.1155	0.80 ± 0.0058	0.50 ± 0.0	1.41 ± 0.0115
Methyl acetylene, % mol	0.42 ± 0.0153	0.71 ± 0.0173	0.52 ± 0.0289	0.31 ± 0.0231	1.60 ± 0.0
***Total Amount***	***0.61 ± 0.0115***	***0.94 ± 0.0981***	***1.30 ± 0.0058***	***0.81 ± 0.0231***	***3.01 ± 0.0115***
**ALCOHOL**					
Methanol, % mol	0.01 ± 0.0	0.05 ± 0.0058	3.71 ± 0.0231	5.43 ± 0.0577	8.40 ± 0.0
Ethanol, % mol	0.11 ± 0.0115	0.21 ± 0.0058	0.86 ± 0.0200	1.61 ± 0.0115	3.73 ± 0.0577
Isopropyl Alcohol, % mol	0.04 ± 0.0100	0.01 ± 0.0	1.03 ± 0.0577	2.43 ± 0.0577	4.20 ± 0.0
N-Propanol, % mol	0.03 ± 0.0	0.09 ± 0.0058	0.83 ± 0.0173	3.07 ± 0.0577	5.83 ± 0.0577
N-Butyl Alcohol, % mol	0	0.05 ± 0.0058	1.43 ± 0.0577	2.65 ± 0.1286	3.31 ± 0.0173
1,2- Isobutenediol, % mol	0	0.02 ± 0.0	0.98 ± 0.0115	1.80 ± 0.0	3.87 ± 0.0608
3-Methyl-2-Pentanol, % mol	0	0	0.22 ± 0.0289	1.62 ± 0.0252	3.17 ± 0.0577
***Total Amount***	***0.18 ± 0.0058***	***0.44 ± 0.0173***	***9.08 ± 0.0058***	***18.61 ± 0.0115***	***32.53 ± 0.0577***
**KETONE**					
Acetone, % mol	0.08 ± 0.0	0.05 ± 0.0058	0.20 ± 0.0	0.80 ± 0.0058	1.83 ± 0.0577
1-Hydroxy-2-Propanone, % mol	0.01 ± 0.0058	0.04 ± 0.0	0.30 ± 0.0	1.20 ± 0.0	2.87 ± 0.0643
2,4-Pentadione, % mol	0.02 ± 0.0058	0.05 ± 0.0058	0.23 ± 0.0577	1.60 ± 0.0	3.08 ± 0.0764
2-Pentanone, % mol	0.13 ± 0.0153	0.11 ± 0.0058	0.70 ± 0.0000	2.43 ± 0.0577	2.80 ± 0.0058
***Total Amount***	***0.25 ± 0.0115***	***0.24 ± 0.0115***	***1.43 ± 0.0577***	***6.03 ± 0.0577***	***10.57 ± 0.0577***
**ACIDS**					
Formic Acid, % mol	0.20 ± 0.0	0.72 ± 0.0058	1.20 ± 0.0	2.43 ± 0.0577	4.57 ± 0.0577
Acetic Acid, % mol	0.10 ± 0.0	0.05 ± 0.0	1.27 ± 0.1155	3.40 ± 0.0	3.83 ± 0.0577
***Total Amount***	***0.30 ± 0.0***	***0.77 ± 0.0058***	***2.47 ± 0.1155***	***5.83 ± 0.0577***	***8.40 ± 0.0***

## Data Availability

Not applicable.
